# Sickle Cell Anaemia Prevalence among Newborns in the Brazilian Amazon-Savanna Transition Region

**DOI:** 10.3390/ijerph16091638

**Published:** 2019-05-10

**Authors:** Rayane Cristina Souza, Pedro Agnel Dias Miranda Neto, Jessflan Rafael Nascimento Santos, Sílvio Gomes Monteiro, Maria Cláudia Gonçalves, Fabrício Brito Silva, Rodrigo Assuncao Holanda, Julliana Ribeiro Alves Santos

**Affiliations:** 1 Biomédica and Mestranda em Meio Ambiente da Universidade CEUMA, São Luís 65075-120, MA, Brazil; naume.rayane@hotmail.com; 2 Faculdade Pitágoras, São Luís 65075-120, MA, Brazil; pedroagnelneto@gmail.com; 3 Laboratório de Geotecnologias, Universidade CEUMA, São Luís 65075-120, MA, Brazil; jessflan@ymail.com; 4 Mestrado em Meio Ambiente da Universidade CEUMA, São Luís 65075-120, MA, Brazil; silvio_gm@yahoo.com.br (S.G.M.); mcgfisio0@gmail.com (M.C.G.); fabricioagro@gmail.com (F.B.S.); 5 Mestrado em Biologia Microbiana da Universidade CEUMA, São Luís 65075-120, MA, Brazil; raholanda@yahoo.com.br; 6 Mestrado em Meio Ambiente e Mestrado em Biologia Microbiana da Universidade CEUMA, São Luís 65075-120, MA, Brazil

**Keywords:** sickle cell anaemia, spatio-temporal distribution, epidemiology

## Abstract

Sickle cell anaemia is one of the most common hemoglobinopathies worldwide and an important public health problem in Brazil. This study evaluated the prevalence of sickle cell anaemia and its traits in newborns from the Amazon-Savanna Transition Region in the state of Maranhão, Brazil. A cross-sectional study was carried out, based on data from neonatal screening tests performed in 2013–2015 in Maranhão. The Hardy-Weinberg theorem was applied to analyse the frequency of expected homozygotes based on HbSS phenotype. A spatial-temporal distribution analysis was performed to delimit the regions with the greatest number of newborn cases with sickle cell anaemia. Of 283,003 newborns, 162 were found to have sickle cell anaemia, while 10,794 had a sickle cell trait, with a prevalence of 0.05% and 3.8%, respectively. The prevalence of expected homozygotes was higher in the North Region and in the state capital of Maranhão. This study may contribute to existing social and public health actions or the creation of new strategies for sickle cell disease in endemic areas in Brazil to improve the quality of life.

## 1. Introduction

Due to its incidence and biopsychosocial complexity, sickle cell anaemia is considered one of the main public health problems worldwide [1]. It originated in Africa and expanded to Saudi Arabia and India. The mutations causing sickle cell anaemia and other hemoglobinopathies were previously non-existent in the Americas; however, the occurrence in Brazil is due to the trade of slaves from the African continent. In Brazil, the distribution of sickle cell anaemia is heterogeneous, with a higher incidence in states such as Maranhão with socio-demographic profiles featuring higher poverty indexes and greater numbers of individuals of African descent [2].

The disease is an autosomal recessive genetic disorder characterised by a change in the beta-globin gene, resulting in the production of abnormal haemoglobin. The haemoglobin change is caused by the substitution of glutamic acid by valine at position 6 of the polypeptide chain. Glutamic acid is a polar amino acid, unlike valine; this change in electrical charge gives the molecule the physicochemical difference responsible for the eructation of red blood cells [3].

Sickle cell anaemia is characterised by haemolysis, chronic and acute inflammation, vaso-occlusive complications, multiple organ damage, and reduced patient survival. The pathophysiology of haemoglobin S (HbS) is complex and influenced by hypoxia, acidosis, and cell dehydration, which result in the polymerization of HbS, leading to erythrocyte deformity. Perturbations in blood flow may increase oxidative stress, causing vaso-occlusive episodes and clinical manifestations such as acute chest syndrome, stroke, priapism, and leg ulcer, among others [4].

Sickle cell trait is the heterozygous form of the HbS gene. It is a condition in which the individual, although not presenting anaemia in routine examination, presents about 40% of haemoglobin S content within erythrocytes. Patients with sickle cell trait are generally asymptomatic, and their quality of life is similar to that of the unaffected population [5].

The screening for anaemia and sickle cell trait in newborns is performed through the neonatal screening that was integrated into the Unified Health System (SUS) in 1992 in Brazil; while mandatory testing was required for all live births, only some states participated. In 2001, GM/MS Ordinance Nº. 822 of Ministry of Health was published, giving rise to the National Neonatal Screening Program, which aims to cover all live births in the Brazilian territory. Since then, all states in the country have become part of the program, which allowed the timely diagnosis of asymptomatic diseases in the neonatal period for early intervention with prophylactic measures and specific treatments to reduce or even prevent physical and mental sequelae [6,7,8].

The treatment of sickle cell anaemia is non-specific; thus, patient survival and quality of life depend on general prevention and treatment measures. The methodologies employed in the screening for sickle cell anaemia serve to identify other types of hemoglobinopathies as well as sickle cell carriers [9,10]. The method used to identify hemoglobinopathies is high-performance liquid chromatography (HPLC).

Considering the importance of this disease in the State of Maranhão, this study aimed to evaluate the neonatal screening program coverage and to characterise the traits and sickle cell prevalence in newborns from the Brazilian Amazon-Savanna Transition Region between 2013 and 2015.

## 2. Materials and Methods

This cross-sectional study was carried out in 283,003 newborns in Maranhão who underwent neonatal screening tests between 2013 and 2015. This is a prevalence study, and the data were analysed on a single occasion. Maranhão has 217 municipalities divided into 19 regional health centres. According to data from the Brazilian Institute of Geography and Statistics (IBGE, 2010), the population of Maranhão is 6,954,036.

The study was based on documentary data sources, in which the results of all neonatal screening tests with FS and FAS standards (homozygosity and heterozygosity for HbS, respectively) were analyzed in the State of Maranhão during the study period. The data analyzed in this article refer to the database of the Neonatal Screening Reference Service of Maranhão, located in the Association of Parents and Friends of the Exceptional (APAE) in São Luís, Maranhão. The Neonatal Screening Reference Service also identifies other variants of hemoglobin and thalassemias, which were not included because they did not fit the objective of this study. The laboratory tests were performed using high-performance liquid chromatography (HPLC).

The initial stage was based on the survey of the number of live births in Maranhão in 2013–2015 from the Information System of Live Births (SINASC) database of the Ministry of Health. The data analyses were based on descriptive statistics, using the calculation of the prevalence coefficient. The prevalence coefficient was calculated by dividing the number of cases by the total number of neonates screened by the National Neonatal Screening Program. The following formula was used to calculate the coverage: (number of children tested/number of children born alive) × 100.

Chi-square tests were used to analyse categorical variables, and the Hardy-Weinberg theorem was applied to analyse the frequency of expected and observed homozygotes for the sickle hemoglobin (HbSS). We used an exploratory analysis of spatial data to analyse the spatiotemporal dynamics of sickle cell anaemia cases in Maranhão in 2013, 2014, and 2015 using ArcGIS 10.2.2 (esri, Redlands, CA, USA).

This research was submitted and approved by the Ethics Committee in Research—CEP of the University CEUMA, under opinion number 2,319,707, in compliance with the requirements of Resolution 466/12 of the National Health Council.

## 3. Results

Between 1 January 2013, and 31 December 2015, 349,176 children were born in the state of Maranhão, 283,003 of which participated in the Neonatal Screening Program. Among the newborns submitted to the test, 162 presented sickle cell anaemia (HbSS) and, in relation to the sickle cell trait, 10,794 had HbAS. The overall test coverage was 81%, ranging from 82.6% in 2013 to 80.3% in 2015 (Table 1).

The distributions of sickle cell trait (10,794) and sickle cell anaemia (162) identified in the neonatal screening per year of research (2013–2015) were: 3811 (35.3%) and 49 (30.3%), 3,683 (34.1%) and 67 (41.3%), and 3300 (30.6%) and 46 (28.4%), respectively. During the study period, there was variability in the numbers of cases of sickle cell anaemia, with the lowest percentage occurring in 2015.

The incidence of sickle cell anaemia among newborns in the State of Maranhão, as determined from samples from children submitted to neonatal screening program from 2013 to 2015, was 57:100,000, while the incidence of the sickle trait was 3814:100,000 (Table 2). Regarding the distribution of the HbSS phenotype among newborns, girls represented 48.8% of the cases, while boys represented 51.2% of the cases diagnosed with sickle cell anaemia (Table 3).

Assessment of the geographical distribution of sickle cell anaemia in Maranhão was evaluated according to the 19 regional health centres in the state and revealed a greater distribution in the capital and North regions. Thirty-two cases were diagnosed in the São Luís region, 15 cases were diagnosed in the Itapecuru-Mirim region, and 15 cases were diagnosed in the Pinheiro region.

**Figure 1:**
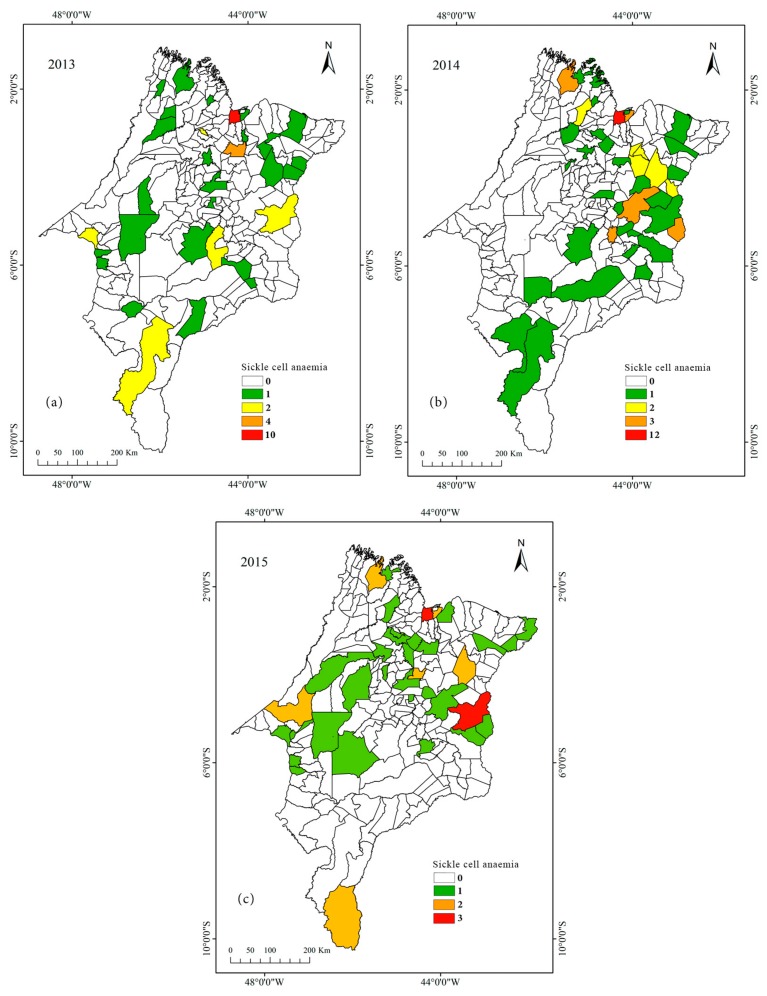
Spatiotemporal distribution of sickle cell anaemia cases in newborns in Maranhão in 2013 (**a**), 2014 (**b**), and 2015 (**c**).

We employed an exploratory analysis to evaluate the spatiotemporal dynamics of sickle cell anaemia cases in the State of Maranhão in 2013–2015. In 2013, the state capital (São Luís) had 10 cases, followed by the municipality of Itapecuru-Mirim with four cases (Figure 1a). In 2014, the municipality of São Luís presented 12 cases, while the municipalities of Codó, Presidente Dutra, São José de Ribamar, Timon, and Turiaçu presented three cases each (Figure 1b). Finally, in 2015, the municipalities of Caxias and São Luís presented three new cases each (Figure 1c).

The observed distributions of sickle cell anaemia expected-genotypes were compared to the expected distributions according to the Hardy-Weinberg theorem. The statistically significant result of χ^2^ adherence tests (χ^2^ = 26.57 *p* < 0.0001) indicated that the distribution of these expected-genotypes did not occur according to Hardy-Weinberg equilibrium. The frequency of the sickle-cell allele (β^S^) in this sample was greater than 1% (1.96%), indicating that the genetic polymorphism seems to be being maintained in the population. The high χ^2^ value (χ^2^ = 26.57 *p* < 0.0001) was due to the deviations determined by the class of expected (β^S^/β^S^) homozygotes (χ^2^ = 25.54), with 53 more cases observed than the 109 cases expected according to Hardy-Weinberg equilibrium. In the two other expected-genotype classes, the observed (272,047 β/β and 10,794 β/β^S^) and expected (271,994.2 β/β and 10,899.6 β/β^S^) cases did not present significant deviations (Table 4).

## 4. Discussion

Maranhão is in Phase IV of the National Neonatal Screening Program, which is responsible for screening for phenylketonuria, congenital hypothyroidism, hemoglobinopathies, and cystic fibrosis. The São Luís’s APAE is responsible for all State Neonatal Screening coverage and offers the complementary service of the Maranhão Hemocenter (HEMOMAR). All 217 municipalities are contracted with PNTN and have 474 collection points, with an average of 2.18 posts per municipality [2].

The results of the present study indicated that the coverage rate was highest in 2013, in which 82.6% of newborns were screened in Maranhão. Another survey carried out in Maranhão by Lopes et al. [11], which screened 99,498 children, showed a PNTN coverage rate of 81.57% in 2008. Another PNTN coverage study in the state of Amapá observed a rate of 31.2%. The deficiency in coverage of Amapá occurred because only 16 municipalities in the state are contracted to the Institute of Hematology and Hemotherapy responsible for the execution of screening throughout the state [12]. In contrast, the coverage rate was 100% of live births in the state of Paraná [13]. During the study period in Paraná, 548,810 newborns underwent the neonatal screening program, and there were 482,094 live births. The total coverage of the state of Paraná likely occurred due to the registration of all hospitals and maternities and the possible inclusion of some border regions of neighbouring countries.

The PNTN coverage rates vary in different regions of the country. Full screening coverage of the population is hampered by socioeconomic and cultural problems, lack of knowledge about the importance of the test, and obstacles related to the movement of the test units. In addition, the coverage of live births may be underestimated since examinations carried out in private networks are not counted. Increased coverage rates should be prioritised, either by the inclusion of the tests performed in the private network or with public actions to meet this need. The early detection of PNTN-treated disorders provides a better prognosis for the disease carrier [14,15].

The appearance of the HbS gene is likely linked to African slavery in Maranhão since the second half of the 17th century [16]. The high prevalence of the disease in the state can be explained by the regular entry of blacks into the states of Grão-Pará and Maranhão, assuring the monopoly of the slave trade, as well as the sale of these slaves to the residents who utilised their labour in the countryside and in the city [17].

Homozygous and heterozygous β^S^ gene are distributed heterogeneously nationwide and are frequently observed in populations with higher proportions of black ancestors. Despite the predominance of these genes in black and mulatto populations, other population studies have demonstrated the increasing presence of β^S^ in Caucasian populations. As a consequence, the North and Northeast Regions, which had the greatest influence of the black race, show higher frequencies of hemoglobinopathies [18].

The prevalence of sickle cell anaemia in this study indicated that one in every 1754 live births had the β^S^/β^S^ homozygous form, supposedly. This high frequency is possibly explained by the large proportion of individuals of African descent in the state of Maranhão [19]. However, one of the limitations of the present study was the impossibility of analysing the percentages of participants with anaemia and sickle cell trait by colour or race due to the lack of this information in the databases.

Although sickle cell anaemia is a non-sex-linked genetic disease, there was a higher prevalence of expected β^S^/β^S^ in male newborns. The present study corroborated data reported in studies carried out at Campana Laboratory and the Assis Chateaubriand Maternity-School in Fortaleza, which analysed blood samples from 1303 and 389 newborns, respectively, and also observed a higher prevalence in male newborns [20,21].

In Maranhão, the frequency of sickle cell trait, as determined by the results from samples submitted to the neonatal screening program, was 3.81% (1:26) in 2013–2015. In Salvador, a survey of 590 newborns reported a 9.8% (1:10) frequency of sickle cell trait, the highest percentage of sickle cell trait in Brazil [15]. A study carried out in Mato Grosso do Sul from 2000 to 2005 of 190,809 newborns submitted to the PNTN reported an average annual HbAS frequency of 1.6%. In 2000, the frequency was 2.4%, the highest in relation to other years, probably due to the low coverage of the PNTN that year [22]. The Neonatal Screening Program in Rio de Janeiro assessed 99,260 samples from newborns born between August 2000 and November 2001. The prevalence of heterozygotes was 4.7% (1:27) [23]. In a study carried out in Santa Catarina, Ellen et al. reported a sickle cell trait frequency of 0.8% among 730,412 children [24].

Lopes et al. analysed neonatal hemoglobinopathy screening data in all municipalities of the State of Maranhão, reporting that 4.9% (1:25) of 99,498 newborns had heterozygous HbS [11]. The results of comparative analysis of the findings of the present study with those of this previous research suggested that the difference in results was related to the size of the studied populations. Lopes et al. analysed samples only from 2008, whereas the present study analysed samples from three consecutive years. Another factor that may have impacted the reduction in the number of cases is prevention actions, such as genetic counselling, directed at parents and individuals identified as having sickle cell trait [25]. Our findings in Maranhão are similar to those in other Northeast states including Bahia [15,26], Rio Grande do Norte [27], and Rio de Janeiro [28], and Minas Gerais [29] in the Southeast, emphasizing the correlation with the history of Brazilian colonisation and reflecting the influx of immigrants of African descent which occurred mainly in these states.

The increased distribution of cases of sickle cell anaemia among newborns observed in the Northeast Region and state capital can be explained by the ethnic composition of these populations and the union of people of the same ethnic groups, which may contribute to a significant increase in the number of homozygous individuals in certain regions of the state. The frequency of expected homozygous individuals indicates that this locus is changing over time in the state of Maranhão. 

## 5. Conclusions

Increased biological and epidemiological knowledge of sickle cell anaemia provides new therapeutic approaches. In addition, the mapping of sickle cell anaemia cases and knowledge about its occurrence in Maranhão may contribute to existing social and public health actions and the creation of new epidemiological surveillance strategies to improve the quality of life of the affected population.

## Figures and Tables

**Table 1:** Neonatal screening coverage and the total number of sickle cell anaemia and sickle-trace cases in newborns in the State of Maranhão, 2013–2015.

9	LB * (N°)	Screening (N°)	Coverage (%)	N° HbSS *	N°HbAS *
2013	115,332	95,329	82.6%	49	3811
2014	117,181	93,956	80.2%	67	3683
2015	116,663	93,718	80.3%	46	3300
**Total**	**349,176**	**283,003**	**81.0%**	**162**	**10,794**

* LB: Live births; HbSS: sickle cell anaemia; HbAS: sickle-trace.

**Table 2:** Prevalence of haemoglobin S in newborns in the state of Maranhão, 2013–2015.

Phenotype	Prevalence (Live Newborns)	N°	%
HbAS	3814: 100,000 (1: 26)	10,794	3.8
HbSS	57: 100,000 (1: 1,754)	162	0.05

HbSS: sickle cell anaemia; HbAS: sickle-trace.

**Table 3:** Distribution of sickle cell anaemia by sex of newborns in the state of Maranhão, 2013–2015.

HbSS	N°	%
Female	79	48.8
Male	83	51.2

HbSS: sickle cell anaemia.

**Table 4:** Distributions of sickle cell anaemia expected-genotypes in newborns identified in the databases and the numbers and percentage of cases according Chi-square tests.

Expected-Genotype	(N) newborns	%	χ^2^ (p)	Alleles
β^S^/β^S^	162	0,06	26.57 (<0.0001)	β = 0.9804
β/β^S^	10,794	3.81		β^S^ = 0.0196
β/β	272,047	96.13		
**Total**	**283,003**	**100**		
